# Trajectories and Resource Management of Flying Base Stations for C-V2X

**DOI:** 10.3390/s19040811

**Published:** 2019-02-16

**Authors:** Silvia Mignardi, Chiara Buratti, Alessandro Bazzi, Roberto Verdone

**Affiliations:** 1 Department of Electrical, Electronic and Information Engineering “Guglielmo Marconi” (DEI), University of Bologna, Viale Risorgimento 2, 40136 Bologna, Italy; c.buratti@unibo.it (C.B.); roberto.verdone@unibo.it (R.V.); 2 National Research Council of Italy (CNR), Institute of Electronics, Computer and Telecommunication Engineering (IEIIT), v.le Risorgimento, 2, 40136 Bologna, Italy; alessandro.bazzi@cnr.it

**Keywords:** cellular-vehicle-to-anything, unmanned aerial vehicles, mobile radio network, connected vehicles

## Abstract

In a vehicular scenario where the penetration of cars equipped with wireless communication devices is far from 100% and application requirements tend to be challenging for a cellular network not specifically planned for it, the use of unmanned aerial vehicles (UAVs), carrying mobile base stations, becomes an interesting option. In this article, we consider a cellular-vehicle-to-anything (C-V2X) application and we propose the integration of an aerial and a terrestrial component of the network, to fill the potential unavailability of short-range connections among vehicles and address unpredictable traffic distribution in space and time. In particular, we envision a UAV with C-V2X equipment providing service for the extended sensing application, and we propose a UAV trajectory design accounting for the radio resource (RR) assignment. The system is tested considering a realistic scenario by varying the RRs availability and the number of active vehicles. Simulations show the results in terms of gain in throughput and percentage of served users, with respect to the case in which the UAV is not present.

## 1. Introduction

The transport system is one of the fields expecting the greatest changes in the next decades. Human-driven cars and trucks will be progressively replaced by connected and autonomous vehicles (CAVs), promising safer mobility, more efficient traffic management, significant pollution reduction, and the availability of new services for passengers.

Focusing on connectivity, for several years IEEE 802.11p and the related standards had been the main solution for the vehicular environment [1]. Starting from 2016, however, 3GPP has introduced the concept of cellular-vehicle-to-anything (C-V2X), which specifically addresses this sector within long term evolution (LTE), 5G, and beyond [2,3,4]. In C-V2X, long and short-range communications are parts of the same framework, with the promise of a single chip-set, a native involvement of vulnerable users, and a long term support from the entire cellular ecosystem.

Regarding the applications envisaged for the vehicular scenarios, often denoted as use cases, a large list has been presented for what are called the Day-1 or Day-1.5, which are in principle supported by both IEEE 802.11p and the first versions of C-V2X and are based on the unidirectional distribution of information to improve context awareness; emergency vehicle warning, stationary vehicle warning, and roadworks warning are just a few examples [5,6,7]. Recently, 3GPP has started a discussion about advanced use cases, where vehicles do not only share basic information about their status through small packets, but also exchange large messages and interact in order to coordinate their actions. Such applications have challenging requirements in terms of wireless capabilities that are hardly guaranteed by current standards [8]. One relevant example is extended sensing [9]; by allowing a collective perception of the environment, it implies the need for data rates that range between 10 Mbps to 1 Gbps within a limited range [8].

Although connected vehicles are often associated with the capability of short-range wireless communications, it is clear that it will take time before all cars are equipped and that the long-range connection guaranteed by cellular base stations must be viewed as a valuable option at least during this transitory. However, providing an ubiquitous and reliable coverage to vehicles is particularly challenging with terrestrial base stations (TBSs), since the density and distribution of vehicles is significantly variable in both time and space, in ways that are not always easy to predict.

In a scenario where the penetration of cars equipped with wireless communication devices is far from 100% and the requirements tend to be challenging for a cellular network not specifically planned for the vehicular scenario, the use of an unmmaned aerial vehicle (UAV), carrying a mobile base station, could be helpful to fill the gap of short-range connections and address unpredictable traffic distribution in space and time. In fact, UAVs might be an efficient complement to traditional TBS [10,11], because they can: (1) fly where TBSs cannot offer good coverage and capacity; (2) satisfy traffic demand when needed, if a proper trajectory is planned; and (3) easily achieve line of sight (LOS) conditions toward vehicles on the ground.

In this work we consider a scenario where C-V2X users are moving in an urban area and request to be served by base stations. A number of TBSs are deployed in the area with limited amount of radio resources (RRs) and one UAV is launched in the area to support the TBSs in the service provision. A joint RR scheduling strategy, between the UAV and the TBSs, is applied and a UAV trajectory is defined according to a heuristic algorithm. The latter is inspired by the cluster-based algorithm defined in [12], where, however, a different weighting cost function was defined to select the trajectory. Results show the improvement of performance in terms of the percentage of successfully served vehicles when using the UAV in addition to TBSs.

The article is organized as follows. Section 2 introduces the main contributions in the field. In Section 3 we explain the system model in all of its aspects, while focusing on the aerial component on Section 4. Then, Section 5 shows the obtained simulation results and Section 6 concludes the article.

## 2. Related Work

Equipping vehicles with wireless communication devices to implement advanced services on the road has been studied for decades and standards today appear mature for large scale deployment [13]. However, given their critical impact on road safety and the need that the various car-manufacturers agree to common technologies, still a small percentage of vehicles is presently connected, normally relying on long-range cellular coverage and providing services that are limited to entertainment [14,15] or vehicle tracking [16].

It is anyway generally believed that CAVs will come to market in the next few years and this represents an enormous opportunity for all companies directly or indirectly involved. In this context, the cellular ecosystem started to think about protocols and services for LTE and 5G specifically devoted to the vehicular environment. Beginning even before first definitions inside the standards in 2016, much effort has thusfar being devoted to C-V2X [17,18]. Most work does actually focus on short-range communications [19,20], which is expected to minimize the delay and guarantee a high spatial reuse, but it is clear that long-range communications will always play a relevant role, especially until the percentage of equipped vehicles will be limited [21,22]. When dealing with long-range communications, however, it appears very challenging to combine a fixed deployment of base stations with stringent requirements of a significantly variable and possibly huge data traffic, which is presumable for vehicles that might be sparse or very dense in different parts of a city or at different times of day [23].

At the same time, UAVs are studied from a number of perspectives and different applications. For example, they are considered in many works as flying base stations. The works [10,11] discuss the integration of drones and aerial platforms in the next generation of networks. They analyze aspects such as coverage, radio access, and backhaul links introduced by multiple drone-cells. The works related to UAVs started by focusing on optimal deployments and moved to trajectory considerations in [24]. In particular, a clusterization algorithm is used with one UAV per cluster. The objective is to study the optimal trajectory and deployment in the internet of things (IoT) uplink communications to minimize the power transmitted by machine nodes and the energy consumed by the UAV. In [25], multiple UAVs are used as relays and they are deployed based on appropriate density and cost functions. These functions determine areas with higher user demand. In [26], how radio-maps can drive UAVs is studied, in order to exploit the environment-dependent path loss in a specific area and take advantage of a better coverage. In our previous works [27,28] we considered issues related to a joint radio resource management (RRM) between the terrestrial base stations and a UAV. However, we had a uniform scenario with a large number of active users; different applications with different demands may require algorithm adaptation and simpler and more practical considerations.

The use of UAVs to support vehicular communications has recently raised significant attention as an efficient, flexible, and limited cost solution when the deployed infrastructure is not sufficient or in case of particular events. The majority of papers investigate the use of UAVs to improve routing in vehicular networks. For example, in [29] a novel reactive algorithm exploiting UAVs as relays is suggested when the capacity or coverage becomes insufficient. In [30], drones are used with store and carry capabilities when disconnections occur due to a low density of vehicles. Also in [31], routing is addressed, with the use of game theory to predict disconnected segments where UAVs should be moved. In [32], some vehicles are assumed to be equipped with drones that can fly within a given control range and act as a relay of a multi-hop route or might carry the data directly to the intended receiver. Throughput maximization under delay constraints is instead addressed by the routing algorithm proposed in [33].

In [34], the authors assume drones deployed over a highway and acting as (IEEE 802.11p based) road side units (RSUs) and evaluate the density of UAVs required in order to make vehicles delivering their data respecting a delay constraint with a given probability.

A broader view about using UAVs in vehicular scenarios is provided in [35,36]. In [35], various possible applications are discussed, including flying accident report agents, flying RSUs, flying speed cameras, flying police eyes, and flying dynamic traffic signals. In addition, some simulations are reported assuming a number of UAVs statically deployed as RSUs to improve the coverage of hazardous areas where accidents occur with some probability. A list of issues and advantages deriving from the use of drones in the vehicular scenario is instead provided in [36]. The same paper shows some simulation results in a highway scenario, quantifying the improvement in terms of throughput and delay derived from the deployment of UAVs, without reference to a specific use case.

Testbeds with few devices have been also implemented in [37,38]. In [37], experiments with two UAVs and three vehicles forming a platoon are conducted, where the drones are exploited to monitor the surrounding territory where the convoy is moving. In that case, IEEE 802.11a and ZigBee are adopted for data and control messages, respectively. Preliminary results demonstrate the feasibility to use drones to cooperate with vehicles. Drones acting as relays for vehicle-to-vehicle (V2V) exchanges are tested in [38], in a hilly area where the LOS is often impaired. Adopting low cost communication devices operating at 5 GHz with omnidirectional antennas, a distance higher than 2.5 km is demonstrated.

In the majority of cases, IEEE 802.11p or another WiFi-related technology is assumed and in none of the mentioned works the cellular standards are explicitly addressed. Furthermore, most results refer to generic applications and the focus is on routing or coverage aspects. Finally, only in a few cases the movements and trajectory of UAVs are investigated. Differently, here we consider a system where an aerial and terrestrial component cooperate to provide the desired quality of service of the extended sensing application, by taking into account all network related aspects. Then, we propose both a dynamic trajectory constructed in real-time and a joint resource allocation between the aerial and terrestrial network.

## 3. System Model

### 3.1. Reference Scenario and Traffic Generated

**Figure 1:**
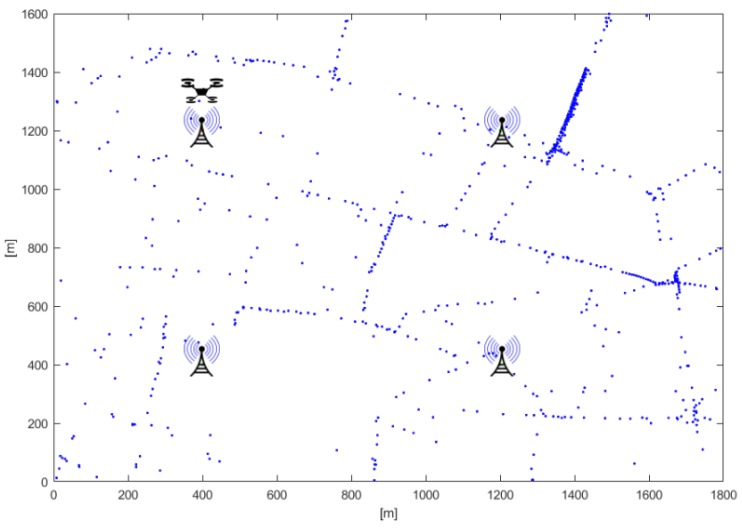
Reference scenario: dots represent vehicles in a given simulation snapshot.

We considered a rectangular area where $N_{\mathrm{BS}}$ sites, with four directional TBSs per site, were uniformly distributed (see Figure 1). The service provider designated a UAV to support the network where and when it is needed. The UAV used, as a starting point for its flight, one of the TBS sites, then, its dynamic trajectory was computed as described in the following.

In the considered scenario, a number of vehicles move according to traffic traces reproduced using the micro-traffic simulator VISSIM, which reproduces in detail the movements of vehicles taking into account the real road network, physical laws and the road rules [39].

Among all $N_{v}$ vehicles, we assumed that a portion $P_{v}$ of them was equipped with C-V2X devices and required the considered service. In the rest of the paper we denote these vehicles as active, with the parameter $P_{v}$ being varied in our simulations to observe the impact of a different load on the network performance.

Further, we set the application requirements to be coherent with the 3 GPP documentation [8] on the extended sensing use case, which defines a range of data rates between 10 Mbps to 1 Gbps. We decided to focus on the downlink only, by setting requirements of either 25 Mb/s or 50 Mb/s. This choice has been made because the downlink becomes a bottleneck for the network when the same sensing information collected from one vehicle has to be forwarded in broadcast to all the interested neighbors.

We assumed that the UAV knew: (1) the position of all vehicles that are under cellular coverage in each time instant and their application requirements; and (2) the pool of RR available to it and the set of RRs used by the TBSs. This can be possible by centralizing these network operations in a network entity that manages both TBSs and UAV through Software Defined Networking (SDN) and network function virtualization (NFV) techniques [27]. Then, the UAV was responsible for: (1) defining its mission and trajectory in real-time; and (2) RR assignment at its side.

As known, UAVs have a limited battery life. Nowadays, a drone flight lasts for 25–30 min [40], but recent developments in technology demonstrate that an endurance of few hours is already possible [41]. In any case, since this is the typical issue of UAVs for the majority of possible applications, a number of recent studies has tackled this problem, such as [42,43]. The former presents a new UAV with an endurance that lasts more than two hours, while the latter studies how to wirelessly recharge drones to make their landing even unnecessary. In this work, we assumed that, when the UAV runs out of battery, it was able to fly back to one TBS site to recharge and be replaced by another one to maintain a seamless operation. Moreover, we considered a small area (few squared kilometers) and we focused only on the operational phase. Actually, it can be noted that in our scenario in the worst case, the UAV has to fly for 721 m; with a speed of 20 m/s it takes only 36 s out of a 30 min flight, thus the recharge phase has a negligible impact on the network performance.

### 3.2. Radio Channel and Physical Layer

As far as the radio interface is concerned, we considered an LTE scenario where vehicles were using C-V2X technology. Table 1 shows the parameters related to the technology and scenario. Both the TBSs and the UAV operated within the same carrier of 2600 MHz and had the same quantity of RRs available.

From the TBSs side, we considered a typical radio channel of an urban scenario, with the propagation exponent, β, of 3.6 [44] and a shadowing variable, $S_{\sigma }$ with log-normal distribution and variance σ=6 dB. Given the equivalent radiated power $EIRP_{\mathrm{tx},\mathrm{TBS}}=P_{\mathrm{tx},\mathrm{TBS}}\cdot G_{\mathrm{tx},\mathrm{TBS}}$, with $P_{\mathrm{tx},\mathrm{TBS}}$ and $G_{\mathrm{tx},\mathrm{TBS}}$ being the transmission power and gain of the TBSs, respectively, the received power $P_{\mathrm{rx},\mathrm{TBS}}$ is computed as

(1) $$P_{\mathrm{rx},\mathrm{TBS}}=EIRP_{\mathrm{tx},\mathrm{TBS}}\cdot {\left(\frac{c}{4\pi \cdot f_{c,\mathrm{TBS}}}\right)}^{2}\cdot {\frac{1}{d}}^{-\beta }\cdot S_{\sigma }$$

where *c* is the speed of light, $f_{c,\mathrm{TBS}}$ is the TBSs carrier frequency and *d* is the link distance. See Table 1 for implementation details.

Differently, the UAV encounters less impairments thanks to its height, and therefore we applied the model of the air-to-ground (ATG) channel proposed in [45]. It mainly consists of extracting the probability of LOS or Non-LOS depending on the height and the angle θ between the ground and the line connecting the UAV to the user. The larger is θ, the closer to one is the probability of LOS. Depending on this, the ATG path loss, PL, is computed. In particular, the LOS probability $P_{\mathrm{LOS}}\left(\theta \right)$ follows

(2) $$P_{\mathrm{LOS}}\left(\theta \right)=\frac{1}{1+a\exp(-b[\theta -a])}$$

where *a* and *b* are parameters defined in [45] that depend on the environment. The ATG channel has for both the LOS and NLOS cases an excessive path loss [45], η, to be added to the free space loss, FSPL. Then, the following equations can be applied

(3) $$PL_{\mathrm{dB}}=FSPL_{\mathrm{dB}}+\eta$$

(4) $$FSPL_{\mathrm{dB}}=20\log\left(d\right)+20\log\left({f_{c,\mathrm{drone}}}_{\mathrm{MHz}}\right)-27.55$$

(5) $$Pr=\frac{P_{\mathrm{tx},\mathrm{UAV}}}{PL},$$

where the subscript dB denotes that the variable is expressed in decibels. In (4), the free-space loss was computed (in dB) as a function of the distance *d* between the vehicle and the UAV and a value of $f_{c,\mathrm{drone}}$ expressed in MHz. Those nodes that have an elevation angle θ below $15^{\circ }$ were not considered in the UAV coverage range as in [45], since their heavy path loss value would not allow a correct signal reception. This implies that the UAV coverage range is limited depending on θ.

We assumed the UAV is equipped with a directional antenna that employs a fixed aperture angle α pointing towards the terrain as in [46]. Assuming an ideal antenna without side lobes and with constant gain, the area potentially covered by the UAV under uniform propagation conditions is thus a circle of radius r=h·tanα. The antenna gain depends on α as well: it is equal to $G_{\alpha }=29,000/{\left(\alpha \right)}^{2}$. We added to such gain 3 dB, to account for a minimum offset for the case in which α is large. Note that, the higher the UAV height, the larger is the UAV covered area and the number of vehicles that it may be able to serve. If, instead, we fix *h*, the larger is α and the larger is the UAV coverage range, but the smaller is the antenna gain.

The terrestrial and aerial components compute the signal to noise ratio (SNR) and signal to interference ratio (SIR) of the different links to take decisions on RRM. In order to achieve a satisfactory quality of service, SNR and SIR for the single vehicle *v* have to overcome a minimum threshold of $SNR_{\min}$ = 10 dB and $SIR_{\min}$ = 2 dB, respectively. The SNR and SIR are computed as

(6) $$SNR_{v,n}=\frac{P_{r\{v,n\}}}{2\cdot N_{0}\cdot B_{\mathrm{subc}}}$$

(7) $$SIR_{v,n}=\frac{P_{r\{v,n\}}}{\sum_{j=1}^{N_{\mathrm{TBS}}}P_{r\{j,n\}}}$$

where $P_{r\{v,n\}}$ represents the useful power received at terminal *v* on subcarrier *n*, $B_{\mathrm{subc}}$ is the equivalent bandwidth of one subcarrier, $N_{0}$ is the noise spectral density, $P_{r\{j,n\}}$ is the interfering power coming from interferer *j*, and $N_{\mathrm{TBS}}$ is the number of interfering TBSs.

For the computation of SIR at TBSs, we assumed that the interference coming from the UAV was negligible thanks to the proposed RR scheduling (see later). The computation of throughput for each vehicle followed the link budget considerations. For both the cases of TBSs and UAV, the throughput achieved by user *v*, $T_{v}$, was computed according to

(8) $$T_{v}=\min\left(\frac{B}{N}\sum_{n=1}^{N}c_{v,n}\log_{2}\left(1+X_{v,n}\right),T_{\max}\right)$$

(9) $$X_{v,n}=\min\left(SNR_{v,n},SIR_{v,n}\right)$$

where $c_{v,n}$ equals one when subcarrier *n* is assigned to user *v* and zero otherwise. *N* is the total amount of subcarriers and *B* the bandwidth. Since the throughput was computed with the Shannon formula, we fixed a maximum achievable capacity per link, $T_{\max}$.

## 4. Aerial Operation

The intervention of one UAV is due to an insufficient service quality from the terrestrial network, resulting in a low number of served vehicles.

### 4.1. Radio Resource Scheduling

The RRM is performed by a high level entity that jointly supervises both the TBSs and UAV (e.g., an SDN controller). Indeed, since the TBSs and the UAV share the same RRs pool (by working on the same carrier frequency with the same bandwidth), a joint scheduling is necessary. For any vehicle that can be served by both the TBSs and the UAV, the one providing the highest throughput is chosen. Algorithm 1 shows the network implementation of RRM, which is executed every second. 

**Algorithm 1:** Radio resource scheduling.**Data**: UAV and TBSs positions, vehicle positions and data-rate demand, β, σ, ATG channel,   $SNR_{\min}$, $SIR_{\min}$, TBSs and UAV resource pool capacity **Result**: Set of served and set of unserved vehicles

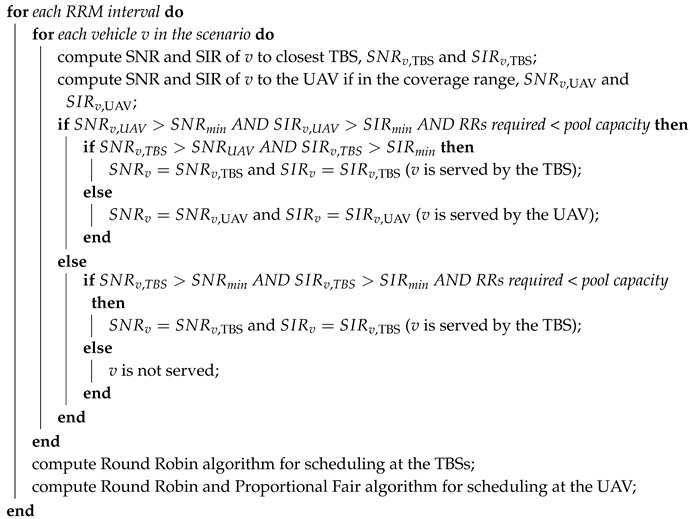



At first, the values of SNR and SIR of a vehicle towards its closest TBS are computed; the same computation is repeated for the link of the vehicle with the UAV, if it is in the coverage range. If both values of SNR and SIR are above the corresponding threshold, the interested base station checks if the vehicle request does not exceed the RRs remaining capacity. Then, when these three requirements are satisfied, a vehicle can be considered served. If the user is in the coverage range of the UAV, it will be served by the station providing the higher SNR value between the TBS and the UAV. In this way, the UAV serves vehicles that would have not been satisfied by the terrestrial network, and improves the service quality of the others by releasing the corresponding RRs.

The scheduling at the TBSs followed a first-come first-served fashion in case not enough resources were available for all the vehicles asking for the service. A Round Robin algorithm was used for resource assignment. In contrast, the UAV was able to employ a different method, thanks to the knowledge of the traffic demand such as all users positions, link quality and service requirements of vehicles: it first served the vehicles with higher SNR. At the UAV, Round Robin was followed by a Proportional Fair algorithm for RRs assignment. In this way, it gained a better efficiency in the service provision.

Since a reuse factor of one is assumed, the TBSs and UAV shared the same pool of RRs. For this reason, in order to avoid high interference levels, the UAV was programmed to reuse only part of the resources. Each time the scheduling was computed, the RRs available for the UAV are the ones not used by the TBSs in its coverage area. Therefore, the UAV had to compute in real-time its RR pool capacity, because it dynamically changed with the UAV movements. In this way, the co-channel interference that the UAV can cause to the vehicles served by a TBS can be neglected and it does not influence the TBS normal operation. In fact, if the vehicle served by a TBS was inside the UAV coverage range (determined by the ATG channel model), its RRs were not reused by the UAV; instead, if it was outside the coverage range, the signal received by the UAV was negligible [45].

### 4.2. UAV Trajectory Planning

The design of the UAV trajectory followed a number of rules to ensure an efficient operation. In this work we started from the approaches of [12,47], and we revised them to adapt to the vehicular scenario. In particular, the path planning was defined according to a number of factors: the position of vehicles, their application requirements, the traffic density and the sum throughput estimation based on the resource pool capacity.

Algorithm 2 represents the protocol implemented for the UAV trajectory. Let us assume it is located in *Q* (*x*, *y*, *h*) at a given instant *t*, when a new flight direction has to be chosen. In our case, the flight lasts 10 minutes. The UAV knew, through a network controller, the updated information on the positions and traffic demand of the vehicles not served yet. Based on this:

- the vehicles out of TBSs service were grouped in *K* clusters, where $K=P_{v}\cdot 100$ (e.g., 10 clusters for 10% of active vehicles, 20 clusters for 20%, etc.) using the centroid-linkage UPGMC algorithm [48];
- for each cluster (i=1, *…*, *K*), its central point, the centroid, was computed;
- for each centroid (i=1, *…*, *K*), a cost function, $C_{i}$, was computed (see below);
- the centroid having the smallest cost value was selected as the next stop and its distance from *Q* is denoted as $d_{k}$;
- the UAV started flying in the direction of the chosen centroid along a segment path and reached the centroid in $d_{k}/s$ seconds, where *s* is the UAV speed;
- during its entire flight, the UAV served all vehicles encountered in its coverage area.

Now, to pursue system efficiency, the cost function design should include parameters such as the UAV energy consumption, its RR availability and the number of users that could be served in one spot. For these reasons, the travel distance, the estimation of network throughput and the density of vehicles are the factors affecting the cost function, which is defined as

(10) $$C_{i}=\frac{d_{i}}{d_{\max}}\cdot \frac{\delta_{\min}}{\delta_{i}}\cdot \frac{S_{\min}}{S_{i}}$$

where $d_{i}$ is the distance of the *i*-th centroid to the UAV, $d_{\max}$ is a normalizing factor equal to the distance of the farthest centroid to the UAV, $\delta_{i}$ is the number of vehicles inside the *i*-th cluster, $\delta_{\min}$ is a normalizing factor equal to the smallest number of vehicles inside a cluster, $S_{i}$ is the estimated sum throughput (ST) obtained in the *i*-th centroid, and $S_{\min}$ is the minimum estimated ST among all clusters. The rationale behind the cost function is to jointly give priority to a region near to where the drone is presently, to a cluster with a large number of vehicles, and to an option allowing to offload a high throughput. Each of these factors corresponds to a number between zero and one, and the cost function results from their product.

This approach determines a UAV trajectory made of segments of different length, each of them covered by the UAV at constant speed *v* and fixed height from the ground *h*.

**Algorithm 2:** Unmmaned aerial vehicle (UAV) trajectory definition.**Data**: *Q*, vehicle positions and data-rate demand **Result**: Next trajectory point Create unserved vehicles set V; create cluster set K, create cost function vector C; initialize time instant $t=t_{0}$;

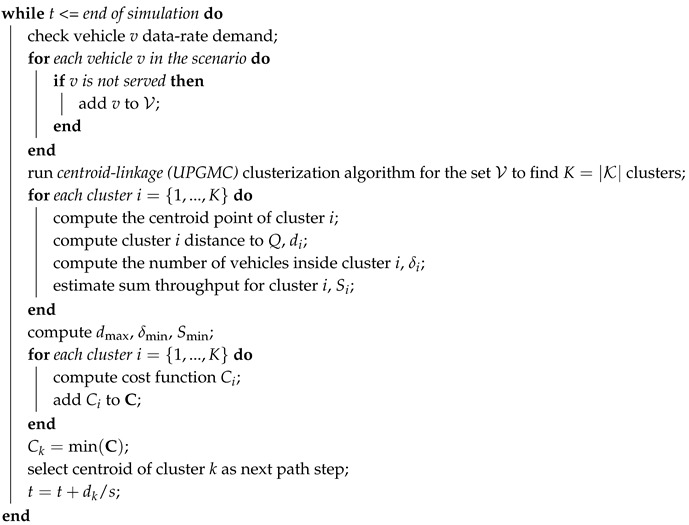



## 5. Numerical Results

We present in this section results with different key performance indicators (KPIs). At first, we study the final sum throughput of the network with and without the support of the aerial component through a single metric to quantify the efficiency of the UAV. Then, we compare the actual percentage of vehicles served directly from the terrestrial network versus the one from the aerial network

We have chosen to test the system under the application requirements of 25 and 50 Mb/s of data rate, in compliance with the already discussed extended sensing.

The first metric of performance assessment of the system is the throughput gain. The gain, *G*, is computed as

(11) $$G=\frac{S_{\mathrm{UAV}}}{S_{\mathrm{TBS}}}$$

(12) $$S_{\mathrm{UAV}}=\sum_{j=1}^{U_{\mathrm{UAV}}}T_{j}$$

(13) $$S_{\mathrm{TBS}}=\sum_{v=1}^{U_{\mathrm{TBS}}}T_{v}$$

where $S_{\mathrm{UAV}}$ and $S_{\mathrm{TBS}}$ represent the ST gained only by the UAV and only by the terrestrial network, respectively, while $U_{\mathrm{UAV}}$ and $U_{\mathrm{TBS}}$ are the total number of users served only by the UAV or only by the TBSs, respectively.

**Figure 2:**
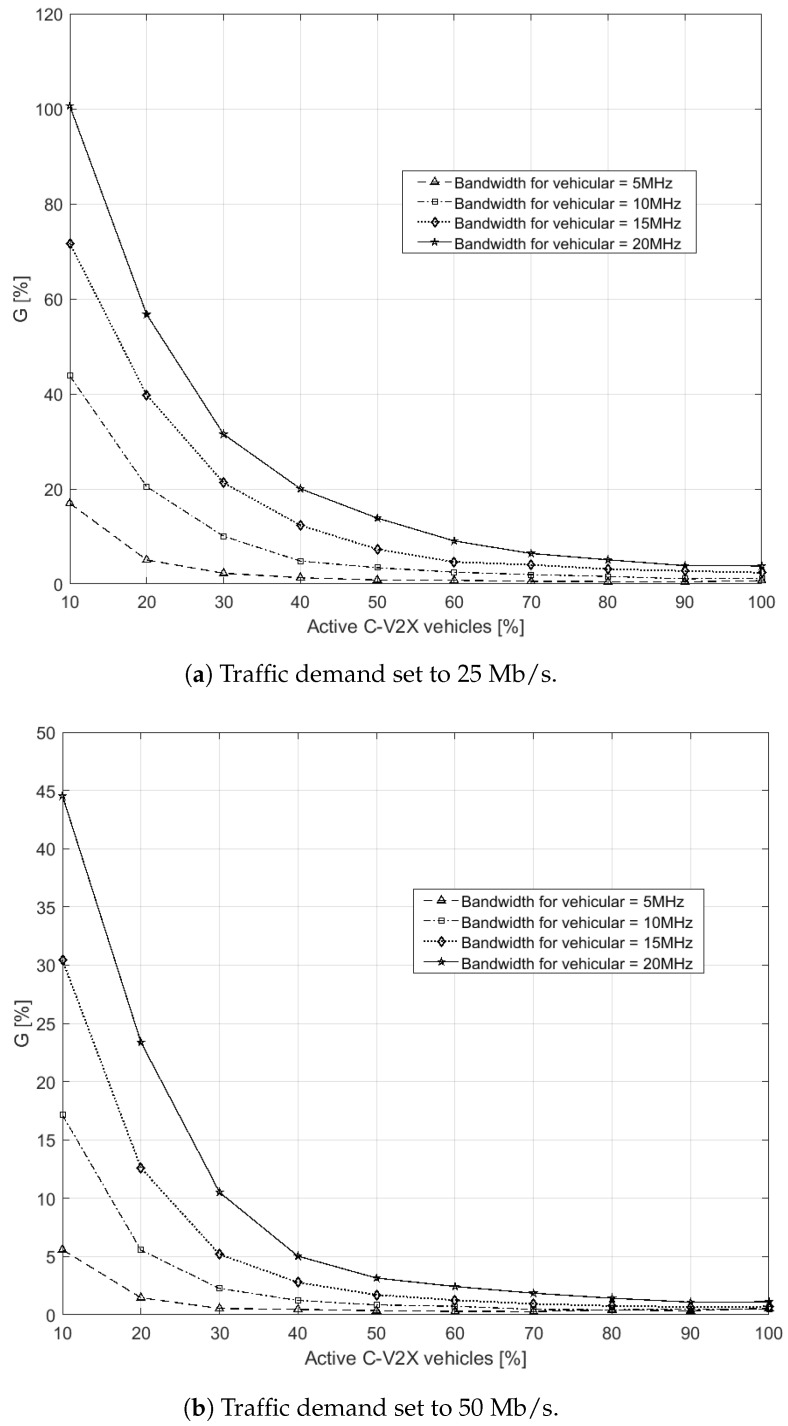
Obtained gain for different bands while varying the number of active vehicles, $N_{v}\cdot P_{v}$, in the network.

Figure 2 shows the gain *G* in percentage while increasing the percentage of active vehicles $P_{v}$. A number of curves were introduced having different bandwidth values, which means a RR pool was changing in size. The bandwidth dedicated to the vehicular service is of 5, 10, 15 or 20 MHz. Figure 2a,b refer to 25 Mb/s and 50 Mb/s, respectively.

**Figure 3:**
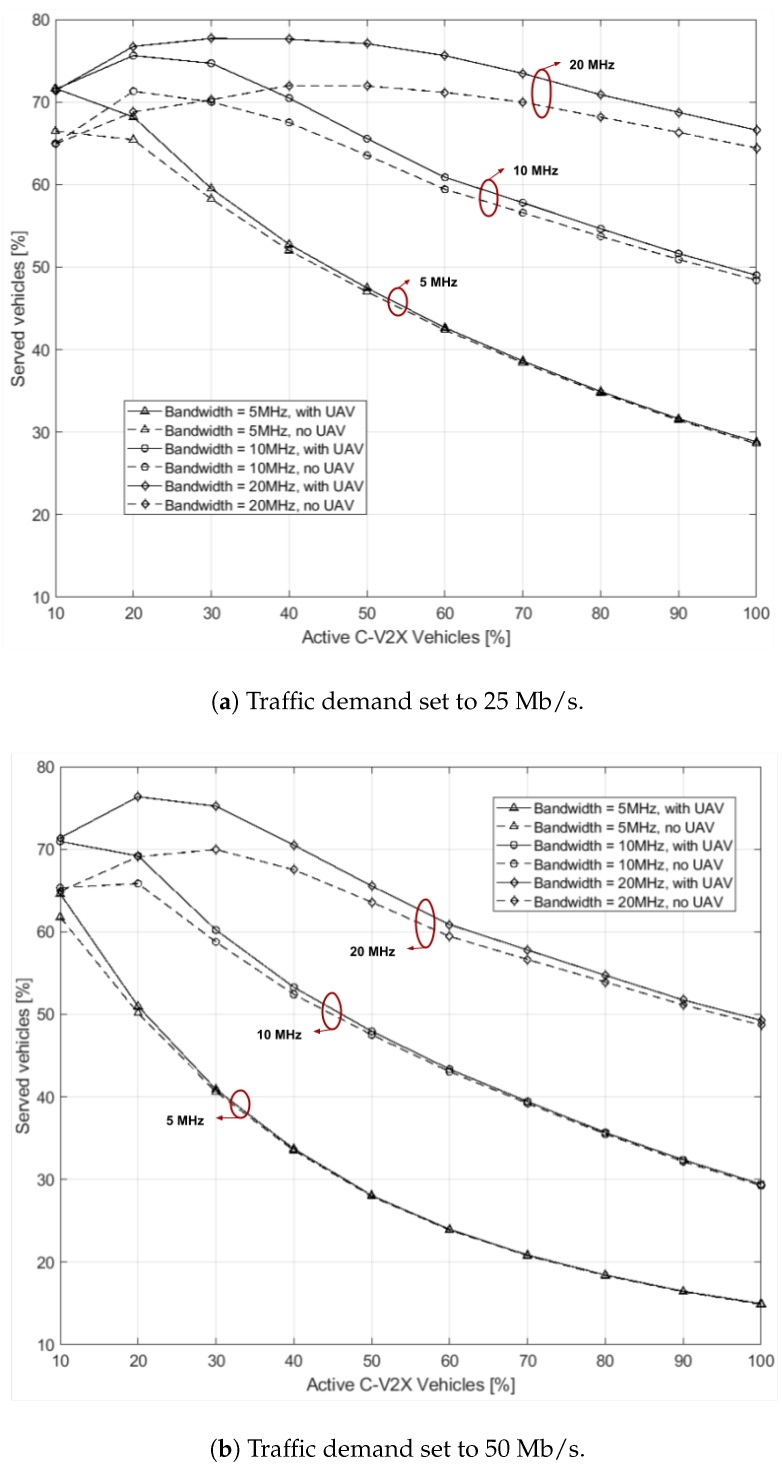
Percentage of served vehicles for different bandwidths and varying the number of active vehicles, $N_{v}\cdot P_{v}$, in the network.

In each curve of Figure 2a,b, the gain *G* had a decreasing trend while increasing the number of active vehicles in the scenario. This can be easily explained by the fact that the TBSs were using a higher number of RRs as the traffic demand increased: due to the common resource pool, the UAV had a lower number of RRs still left available. This network response to an increased offered traffic, stayed the same as the bandwidth (i.e., RR pool capacity) reserved for vehicular applications increases. A larger system bandwidth allowed the UAV to maintain an interesting *G* value close to 10% with a higher number of active vehicles. Moreover, the values of the gain *G* in Figure 2a differ from the corresponding ones in Figure 2b. In the former plot, the values of *G* for the 10% of active vehicles range from a 20% to almost 120%, whereas half of such values can be observed in the latter. This can be explained in terms of availability of resources, too. In Figure 2b the application demand from vehicles is doubled w.r.t. Figure 2a, requiring then double the number of resources from TBSs and the UAV to serve each vehicle. This means that, not only the TBSs employ more resources subtracting them from the UAV pool, but also a lower number of vehicles will be served (see also Figure 3). Furthermore, note that, when considering the transitory in which a few of the travelling vehicles are equipped with C-V2X devices, these results are relevant. In fact, a single UAV was capable of providing up to 100% gain with the proposed approach, even with reuse factor of one. When a larger number of vehicles is equipped with C-V2X services, the probability that two vehicles are close enough to each other and can share useful nearby information with the short range dedicated communication in the 5.9 GHz band increases. For this reason, it is still acceptable that the gain in network throughput offered by the drone remains at 5% to 2% (i.e., in absolute numbers, it is in the order of hundreds of Mb/s).

For the second set, Figure 3 shows the number of vehicles served by the entire network while increasing the number of active users. Two plots are shown when the traffic demand has 25 Mb/s (Figure 3a) and 50 Mb/s (Figure 3b) throughput requirement. Different curves for three different bandwidth sizes of 5, 10 and 20 MHz are shown. For each dedicated band, two curves differentiate the network behaviour with or without the presence of one UAV. A similar trend can be seen for both Figure 3a,b: when more vehicles ask the network for service, it is more probable that TBSs or the UAV have not enough RRs to serve them. If the percentage of satisfied vehicles does not reach 100%, this is due either to SNR or SIR under the respective thresholds. As happened with the gain *G*, the larger is the number of active vehicles, the larger is the probability that RRs available are not sufficient. Also, in both plots of Figure 3, the varying capacity of the UAV RR pool is affecting the performance as in Figure 2: an increasing number of RRs employed by the TBSs limit the UAV efficacy. The UAV, in average, becomes especially effective when the active vehicles are below 50%. Being interested in supporting the network in the initial phase when few vehicles are able to communicate, these results are promising both in varying the bandwidth size and the application data-rate requirements. In particular, with a bandwidth size as large as 20 MHz, the advantage of including one UAV reusing the same RRs as TBSs proves to be an interesting choice even at a later stage. As expected, the different needs of resources in the two cases make the network serve 68% of vehicles in Figure 3a and 50% of vehicles in Figure 3b in the best case (i.e., 20 MHz band) when 100% of them is active.

## 6. Conclusions

In this paper we have introduced the novel concept of UAVs working alongside the terrestrial network to serve vehicles by using the C-V2X technology. We have studied an urban scenario from the network of terrestrial base stations and a UAV perspective. To this aim, we: (1) define vehicular applications requirements compliant to 3GPP documents; (2) apply an RRM technique that ensures network efficiency; and (3) adapt the trajectory design to the specific vehicular use case. Simulation results show that, in particular for the initial phase in which few vehicles are equipped with C-V2X devices and should interface with the LTE network, the proposed system model is promising. A single UAV increases the number of satisfied users of up to 10%. The scenario becomes more critical when an increasing number of vehicles is asking for service and the interference avoidance does not allow the UAV to use all resources. Nonetheless, if the bandwidth reserved for vehicular applications is above 10 MHz and the throughput requirement is not much larger than 25 Mb/s, one UAV can efficiently enhance network performance even when 100% of vehicles are active. The re-use of RRs brings a smaller performance improvement with respect to the opposite case of no re-use. However, it is a significant option to decrease operational costs, especially if intended for small base stations like UAVs that can fly on-demand.

These results encourage us to continue the study of UAVs in vehicular applications, in particular to tackle the connectivity of an increasing number of vehicles. UAVs employed with C-V2X capabilities can be considered as vehicles themselves employing short-range communications. In this way, especially for extended sensing applications, we can envision communications from a vehicle to the UAV to another vehicle in the dedicated 5.9 GHz band, then back to the network (if needed) through LTE. 

## Figures and Tables

**Table 1:** Mobile network parameters.

Parameter Definition	Value
Rectangular area, *A*	1.8 × 1.6 m${}^{2}$
Average number of vehicles in the scenario	600
Number of TBSs in the area, $N_{\mathrm{TBS}}$	16
TBSs equivalent radiated power, $EIRP_{\mathrm{tx},\mathrm{TBS}}$	43 dBm
UAV transmit power, $P_{\mathrm{tx},\mathrm{UAV}}$	20 dBm
Bilateral noise density, $N_{0}$	4 × 10${}^{-20}$ W/Hz
Single carrier frequency on UAV and TBS, $f_{c,\mathrm{TBS}}=f_{c,\mathrm{drone}}$	2600 MHz
Subcarrier spacing, $B_{\mathrm{subc}}$	15 kHz
Number of subcarriers of a resource block	12
Maximum capacity, $T_{\max}$	100 Mb/s
Time slot interval	0.5 ms
Frame time duration	10 ms
Bandwidth of TBSs	[5–20] MHz
Reuse factor	1
UAV speed, *s*	20 m/s
UAV altitude, *h*	120 m
UAV antenna aperture angle, α	120 deg
